# Changes in the BTK/NF-κB signaling pathway and related cytokines in different stages of neuromyelitis optica spectrum disorders

**DOI:** 10.1186/s40001-022-00723-x

**Published:** 2022-06-21

**Authors:** Huimin Qiao, Zhuofeng Mao, Wei Wang, Xin Chen, Suhuan Wang, Haolong Fan, Tianyi Zhao, Huiqing Hou, Mei Dong

**Affiliations:** 1 Department of Neurology, Second Hospital of Hebei Medical University, Shijiazhuang, Hebei 050000 People’s Republic of China; 2 Department of Neurology, Xingtai People’s Hospital, Xingtai, Hebei 054000 People’s Republic of China

**Keywords:** Neuromyelitis optica spectrum disorders, BTK, NF-κB

## Abstract

**Objective:**

Neuromyelitis optica spectrum disorders (NMOSDs) are blindness-causing neuritis; their pathogenesis is still not fully elucidated. Although it has been determined that Bruton tyrosine kinase (BTK) and NF-κB are associated with NMOSD, the changes that occur in different periods remain unknown. The study aimed to demonstrate the changes in the BTK/NF-κB pathway and related chemokines in different stages of NMOSDs.

**Methods:**

A total of 32 patients with NMOSD were selected as the experimental group, and 32 healthy volunteers were included in the control group. In this study, the BTK/NF-κB pathway and related chemokines in the cerebrospinal fluid and peripheral blood samples of patients with NMOSD were analyzed in the acute or remission phase.

**Results:**

BTK, NF-κB, PI3K, IKK, CXCL2, and CXCL12 levels in the NMOSD group in the acute or remission phase were significantly higher than those in the control group (*p* < 0.05).

**Conclusion:**

The BTK/NF-κB pathway plays a vital role in the progression of NMOSD pathology. Our results shed light on its important role as a therapeutic target for NMOSD.

## Introduction

Neuromyelitis optica spectrum disorders (NMOSDs) are autoimmune inflammatory demyelinating diseases related to humoral immunity in the central nervous system (CNS) [1]. A specific antibody to astrocyte (AS), aquaporin-4 (AQP4), is detected in 60–80% of patients with NMOSD, which is a major differentiating feature that differentiates NMOSD from multiple sclerosis (MS). NMOSDs depend on complement and antibody-mediated cytotoxicity for their pathogenesis [2]. These autoantibodies bind to AQP4 expressed by ASs and initiate inflammatory cascades facilitating astrocyte necrosis.

Peripheral blood B lymphocytes produce AQP4 antibodies (AQP4-Ab). These B lymphocytes activate cell cascade reactions via B-cell receptors (BCR) on the cell membrane and downstream signaling pathways, leading to cell proliferation and antibody production [3]. Bruton tyrosine kinase (BTK) is an essential kinase in the BCR signaling pathway [4]. It mediates multiple downstream signaling pathways, such as phosphatidylinositol 3-kinase (PI3K), inhibitor of nuclear factor kappa B Kinase (IKK), and nuclear factor kappa B (NF-κB), thereby participating in cell proliferation and differentiation [5]. BTK, PI3K, IKK, and NF-κB are the central hubs that connect proinflammatory signaling pathways for survival, proliferation, and cytokine production. In autoimmune diseases, such as systemic lupus erythematosus (SLE), rheumatoid arthritis (RA), MS, and MS animal models (e.g., EAE), targeted inhibition of BTK and PI3K significantly affects the production of cytokines and autoantibodies, which effectively inhibits CNS inflammation and disease progression [6, 7]. Inhibiting IKK can downregulate the expression of NF-κB and reduce the production of proinflammatory factors [8]. However, the molecular mechanism of these kinases in NMOSDs remains unclear.

Chemokine ligand protein 12 (CXC-motif chemokine ligand 12, CXCL12) exhibits chemotactic activity for monocytes, neutrophils, and early B-cell precursors [9]. CXCL12 binds to CXC-motif chemokine receptor 4 (CXCR4), which is located on the membranes of B lymphocytes, and possibly activates BTK and PI3K, mediating the proliferation and adhesion of B lymphocytes. Chemokine ligand protein 2 (CXC-motif chemokine ligand 2, CXCL2) can also mediate innate cell immunity by interacting with chemokine ligand receptor 2 (CXC-motif chemokine receptor 2, CXCR2) to promote the migration of neutrophils [10]. Further, CXCL2 can interact with NF-κB and promote inflammation [11]. To date, no changes have been described for CXCL12 or CXCL2 in NMOSD.

In this study, the expression of BTK, PI3K, IKK, and NF-kB as well as chemokines CXCL12 and CXCL2 in the peripheral blood samples of patients with NMOSD and healthy controls was investigated. We explored the changes and possible mechanisms of the NF-kB signaling pathway and related chemokines in the pathogenesis of NMOSDs.

## Materials and methods

### Patients and examinations

From January 2018 to January 2019, 32 patients with optic NMOSDs who were treated at the Department of Neurology in the Second Hospital of Hebei Medical University were selected and categorized into acute (24 cases with an average age of 40.29 ± 15.27 years) and remission (8 cases with an average age of 44.63 ± 12.37 years) phases. The study protocol has been approved by the ethics committee of the Second Hospital of Hebei Medical University. Patients in the acute phase met the following criteria: ① the presence of new neurological symptoms and signs with a duration of > 24 h without remission, and the score of the Expanded Disability Status Scale (EDSS) was increased by ≥ 1 point; ② before the blood sample collection (< 24 h), no immunosuppression or immunomodulation treatment was performed; and ③ the absence of other infectious or autoimmune diseases. Patients in the remission phase met the following criteria: ① no new neurological symptoms or signs appeared before the blood sample was collected; ② patients with NMOSD were previously diagnosed; and ③ the absence of other infectious or autoimmune diseases.

At baseline, several characteristics, such as sex, age, disease course, number of relapses, and the EDSS score, were included. The international standards for NMOSD revised in 2015 were used for diagnosis.

In parallel, 32 healthy volunteers with the same baseline characteristics (29 women, 3 men; with an average age of 41.59 ± 14.28 years) were included in the control group. Comparison of general data among the three groups is shown in Table 1. All participants provided written informed consent.

**Table 1:** General information on research objects

	NMOSD acute phase	NMOSD remission phase	Control group	*P* value
Number of cases	24	8	32	
Gender (women/men)	23/1	6/2	29/3	>0.05
Age (means ± SD)	40.29 ± 15.27	44.63 ± 12.37	41.59 ± 14.28	> 0.05
EDSS score median (inter-quartile range)	3.5 (2)	2 (2.38)	–	> 0.05
Relapsing events median (inter-quartile range)	1.5 (4)	1.5 (2)	–	> 0.05
AQP4-Ab (positive)	84.21%	66.67%	–	> 0.05
Segment of the spinal cord involved (means ± SD)	5.85 ± 4.78	4.80 ± 2.28	–	> 0.05

### AQP4-Ab was measured using cell-based transfection immunofluorescence assay (CBA)

The serum AQP4-antibody was measured using a microscopic live CBA method as described previously [11, 12].

### BTK, NF-κB, PI3K, IKK, CXCL2, and CXCL12 mRNA levels were measured using reverse transcription-polymerase chain reaction (RT-qPCR)

Total mRNA extracted from PBMCs using the TRIzol reagent (DP405-02, Tiangen Biochemical Technology Co., Ltd., Beijing, China) was reverse transcribed into cDNA using PrimeScript™ RT reagent kit (Takara) for quantitative PCR (ABI7500, USA) in the presence of a fluorescent dye (SYBR Green I; Cwbio). Then, mRNA was quantified after normalization to 18srRNA expression. RT-qPCR was used to analyze the levels of BTK, NF-κB, PI3K, IKK, CXCL2, and CXCL12 mRNA. Primer sequences are presented in Table 2.

**Table 2:** Primers for RT-qPCR

Primers	Forward (5ʹ-3ʹ)	Reverse (5ʹ-3ʹ)
Btk	AACTACCTGAGGGAGATGCG	GGTGAAGGAACTGCTTTGACT
PI3K	CATCTGGGAAGTAACAACGC	AGCAGGAAGTCAACCACAAC
Ikk	TCCTACAGTGACAGCACAGAGATG	ACCAAACAGCTCCTTGAGCAC
NF-κB	GGGGACTACGACCTGAATG	GGGCACGATTGTCAAAGAT
CXCL2	GAACATCCAAAGTGTGAAGGTG	CAGTGTGGCTATGACTTCGGT
CXCL12	GGCTCCTGGGTTTTGTATTCTCTG	CGTCCCTCTTGGTGGCTCTCC
18srRNA	GTAACCCGTTGAACCCCATT	CCATCCAATCGGTAGTAGCG

### CXCL2 and CXCL12 levels were measured using enzyme-linked immunosorbent assay (ELISA)

To assess the levels of CXCL2 and CXCL12, venous blood samples were obtained from all the patients before treatment. We used the human growth regulating oncogene β (GROβ/CXCL2) ELISA kit (Elabscience) and the CXCL12 quantitative analysis ELISA kit (Qiaoyi Biotechnology Co., Ltd. Shanghai, China) by following the manufacturer’s instructions.

### Statistical analysis

The results are expressed as mean ± S.E.M. Statistical analysis was performed using SPSS software version 23.0. The data were not normally distributed, and the median (inter-quartile range) was used to uniformly express the data. The frequency and composition ratio were used to describe the general demographic characteristics of patients. The Mann–Whitney *U* test was used to perform intergroup tests on the count data, and the Spearman’s rank correlation coefficient was used to examine data correlation. According to *α* = 0.05, *p* < 0.05 was considered statistically significant.

## Results

### Aquaporin-4 antibody test results

**Fig.1:**
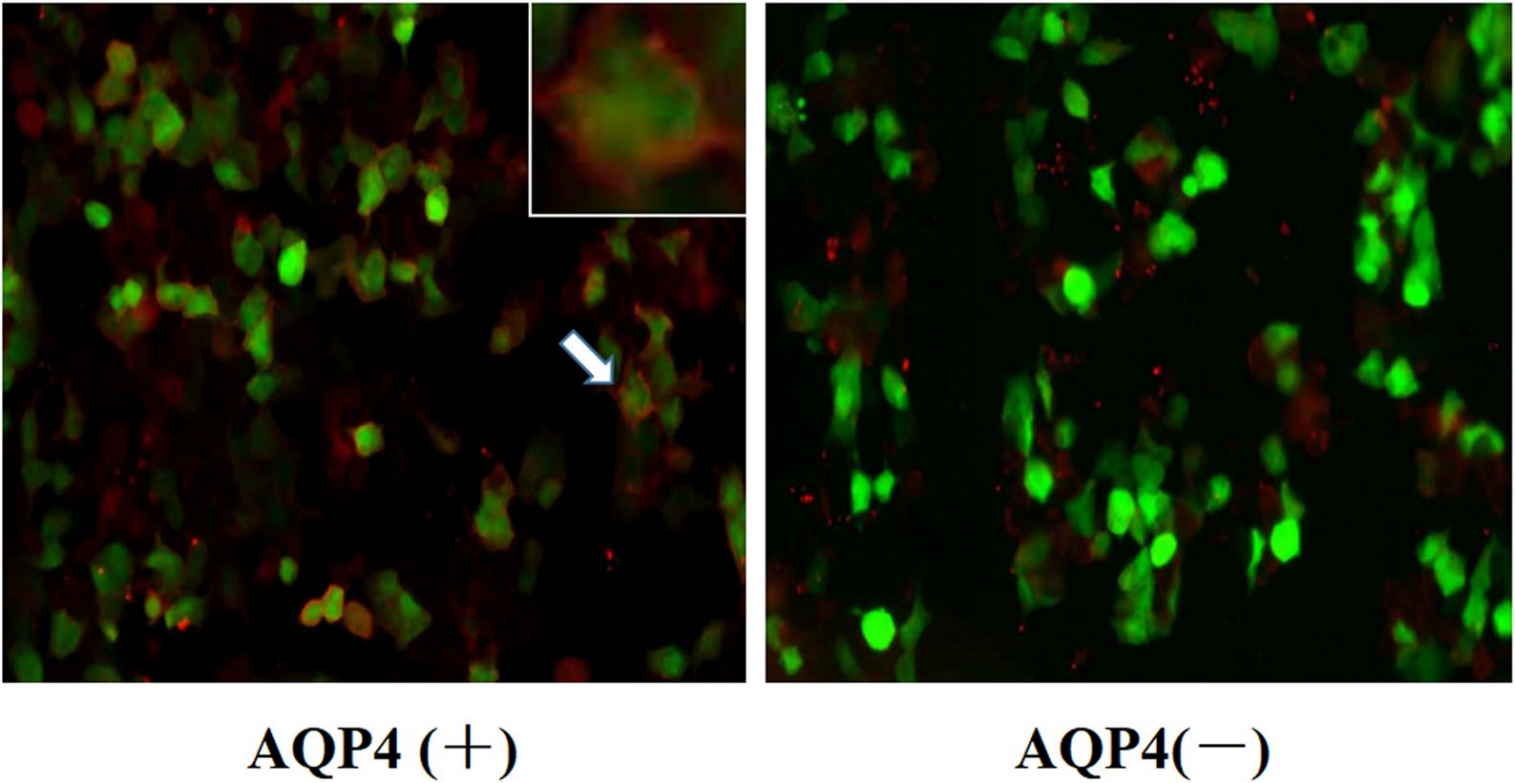
Positive and negative staining patterns in AQP4 expressing cells

The results showed that 26 of the 32 patients with NMOSD were positive (81.3%), and 6 patients were not evaluated using AQP4-Ab (Fig. 1). A comparison of general data among the three groups is shown in Table 1.

### Comparison of BTK, PI3K, IKK, NF-κB, CXCL2, and CXCL12 mRNA levels between NMOSD acute and control groups and those between acute and remission phases.

**Fig.2:**
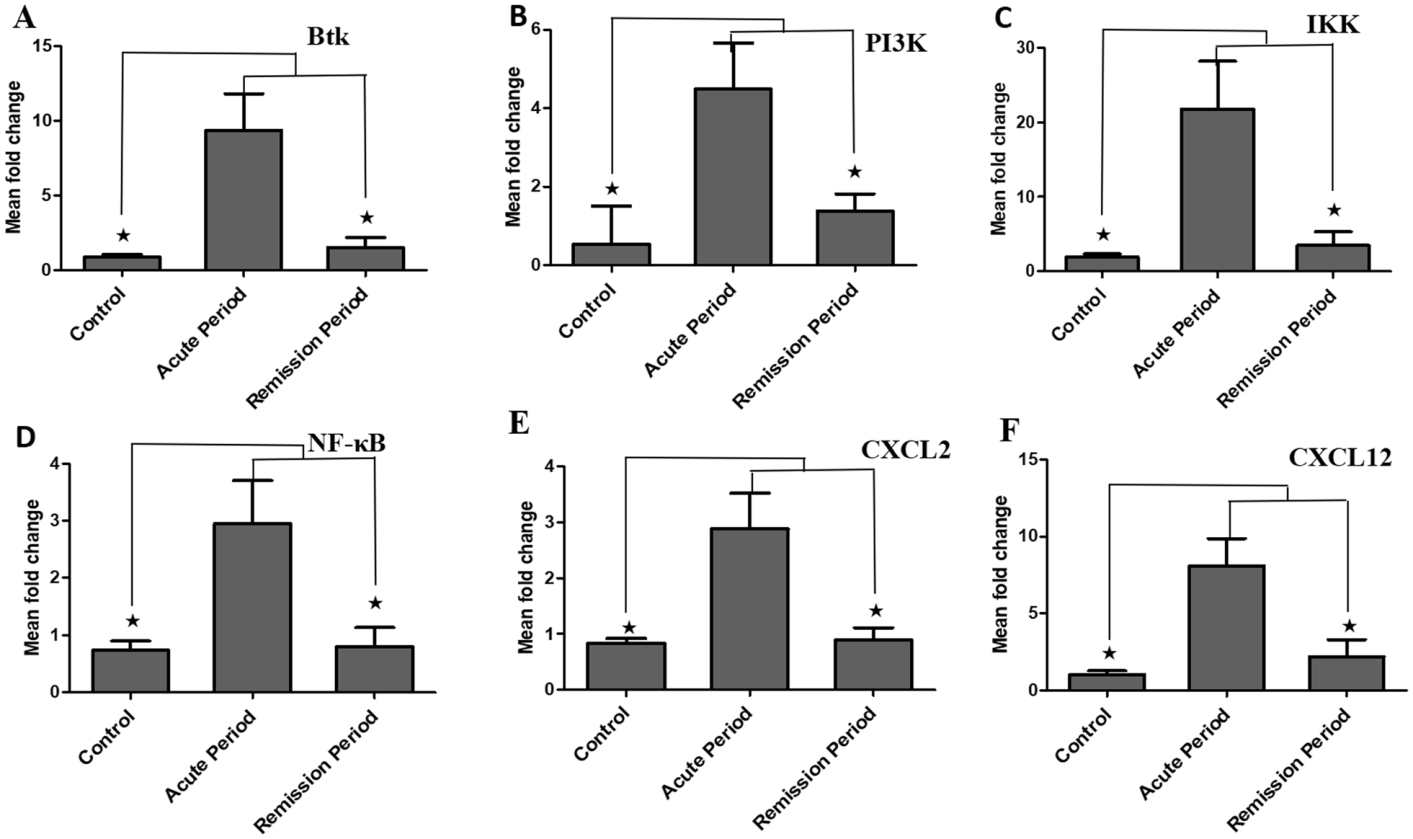
BTK, PI3K, IKK, NF-κB CXCL2, and CXCL12 mRNA levels in the NMOSD group. mRNA expression of BTK, PI3K, IKK, NF-κB and CXCL2, CXCL12 in the acute and remission groups (**A**–**F**). ^★^*p* < 0.05 vs. Vehicle group; ^★^*p* < 0.05 vs. remission group

First, we compared the expression levels of kinases and inflammatory chemokines in mononuclear cells between the patients in the NMOSD and control groups. BTK, PI3K, IKK, NF-κB, CXCL2, and CXCL12 mRNA levels in the acute group were significantly higher than those in the control group (*p* = 0.000, *p* = 0.000, *p* = 0.000, *p* = 0.000, *p* = 0.000, and *p* = 0.000, respectively; Fig. 2A–F). BTK, IKK, NF-κB, CXCL2, and CXCL12 mRNA levels of the patients in the acute group were significantly increased compared with those in the remission group (*p* = 0.004, *p* = 0.012, *p* = 0.009, *p* = 0.013, and *p* = 0.020, respectively; Fig. 2A–F). However, the PI3K level was significantly higher in the remission group than in the control group.

### Serum levels of CXCL2 and CXCL12 significantly increased in the NMOSD group compared with the control group

**Fig.3:**
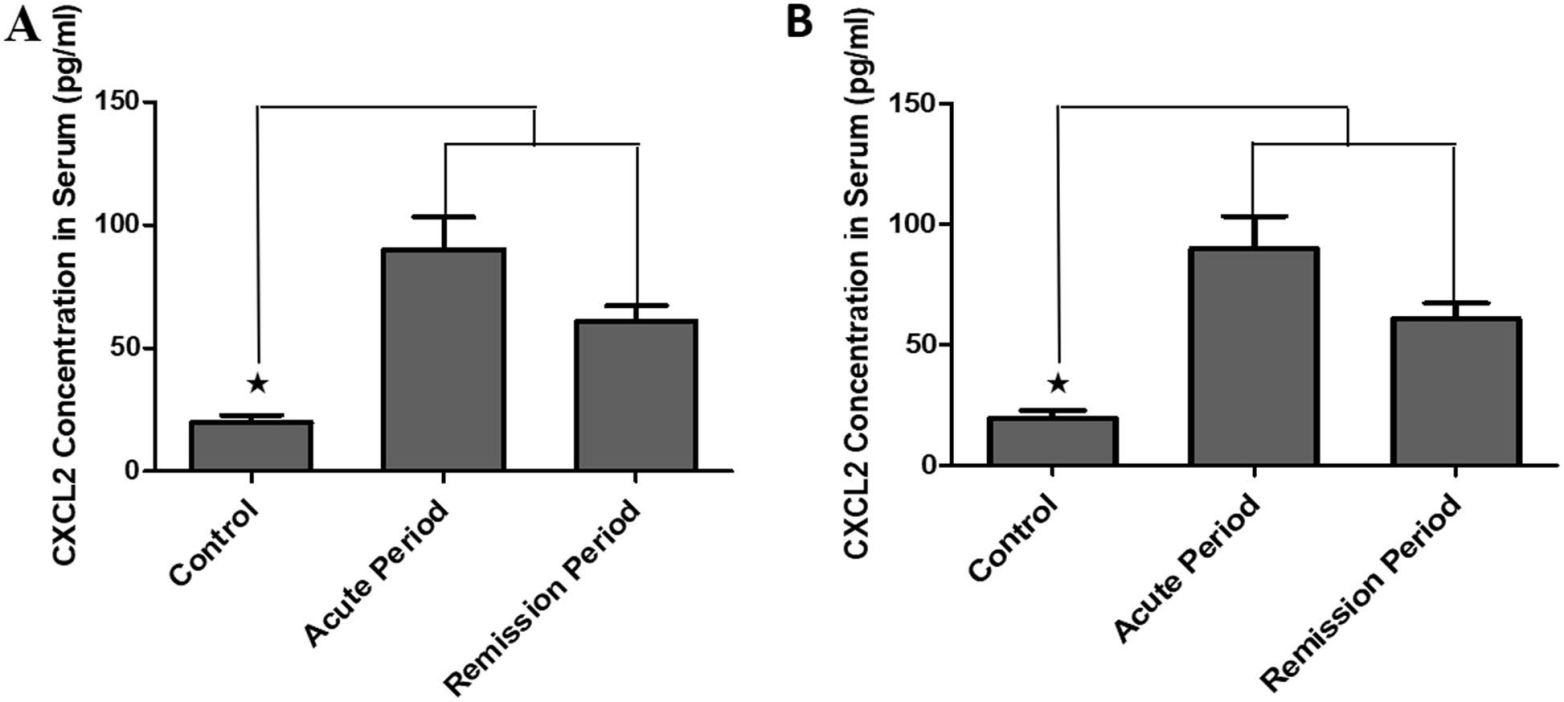
Protein levels of *CXCL2* and *CXCL12* in the NMOSD group. Protein expression of *CXCL2* and *CXCL12* in the acute and remission groups (**A**–**B**). ^★^*p* < 0.001 vs. Vehicle group; ^★^*p* < 0.001 vs. remission group

The serum levels of CXCL2 and CXCL12 in the acute and remission groups were significantly higher than those in the control group (*p* < 0.001, Fig. 3A–B).

## Discussion

NMODs, a spectrum of inflammatory and demyelinating disorders in the CNS, are mainly caused by abnormal humoral immunity. In the acute phase, the important kinase BTK and its downstream NF-κB signaling pathway related to B lymphocytes were significantly activated, and the expression of chemokines (CXCL2 and CXCL12) was significantly increased compared with the control group. However, only PI3K transcription was higher in the remission period compared with the control group, indicating that immune function remains abnormal during the NMOSD remission period.

Systemic autoimmune disorders are complex heterogeneous chronic diseases that involve the interaction among different immune cells. BTK is a crucial signaling protein that directly links BCR signals to B-cell activation, proliferation, and survival. BTK is expressed in B cells as well as myeloid cells and is involved in different signaling pathways that drive autoimmunity. BTK can promote the response of BCR to antigen binding and stimulation through CD40, Toll-like receptor (TLR), Fc receptor (Fcr) and chemokine receptor (CCR) activate the downstream NF-κB pathway, producing cytokines and antibodies, and mediating cell proliferation, differentiation, activation and immune function [12]. In SLE patients, increased BTK expression in peripheral blood was associated with lupus nephritis [13, 14]. BTK protein was elevated in B cells of patients with primary Sjogren’s syndrome (PSjS) [15]. Jansson et al. reported that XID mouse models harboring BTK mutations showed lower chances of developing arthritis in the absence of BTK [16]. Abnormal BTK activity may result in abnormal B-cell activity and subpopulation distribution. In a study of B-cell granulomatous polyangiitis, B cells from patients in the active phase were more sensitive to B-cell receptor (BCR) stimulation than those from patients in the remission phase. At the same time, phosphorylation of BTK in vivo was obviously increased [17]. However, the changes of BTK in NMOSDs were still unclear. In this study, the changes of the BTK signaling pathway and related cytokines in different stages of NMOSDs were investigated, which is the first study focused on these factors in NMOSDs. We found that BTK and related cytokines were significantly increased in acute phase of NMOSDs. BTK inhibitors, including acalabrutinib, BMS-986142, elsubrutinib, evobrutinib, fenebrutinib, poseltinib, rilzabrutinib, spebrutinib and tirabrutinib, have been studied in clinical trials of systemic autoimmune diseases. Most of them are used for treatment of SLE, primary SjS, RA and MS. In vitro experiments have proven that blocking BTK had a significant effect on the production of cytokines, the formation of plasma cells and the production of autoantibodies [17]. The highly selective BTK inhibitor ibrutinib has shown therapeutic effects in MS patients, who had significantly fewer intensive lesions than those treated with placebo [18]. BTK inhibitors may be also a potential therapeutic target for NMOSDs, and the effect of different BTK inhibitors in NMOSDs will be further studied in our following experiments.

PI3K, IKK, and NF-κB are involved in several important signaling pathways downstream of BTK and play significant roles in the development of autoimmune diseases. PI3K is activated by BTK and non-receptor type tyrosine protein kinase (LYN) to produce “second messenger” PIP3, which mediates BTK dimerization on the cell membrane and other key steps for further recruitment and activation [19]. PI3Kγ and δ mediate cell innate immunity and acquired immunity, respectively [20], and play a key regulatory role in various functions, such as cell survival, signal transduction, and membrane transport control [21]. Inhibition of PI3Kδ and PI3Kγ could reduce disease progression in SLE mouse models [22]. In the EAE model, the severity of symptoms was attenuated in mice lacking PI3Kδ subtypes [23]. Further, the inhibition of PI3Kγ could effectively suppress CNS inflammation, alleviate disease progression [24], and reduce nerve demyelination. Target B-cell signaling via BTK, PI3K, or Janus kinases, which are believed to be involved in MS etiology, may provide novel mechanisms to better clarify the humoral immune pathogenesis of MS [25]. Our research found that the expression of PI3K in patients with NMOSD was significantly increased compared with the normal control group, and this result was found in the acute as well as remission periods, suggesting that patients in the remission period are still in an abnormal immune state.

Transcription factor NF-κB exerts multiple immune regulation functions and promotes inflammatory response by inducing the expression of genes encoding cytokines, preimmune response proteins, and antiapoptotic molecules. NF-κB is a key regulator of immune response, inflammation, and cell survival. IKK is upstream of the key link triggering NF-κB activation [26]. PI3K activates IKK through Akt, degrades IκB, mediates NF-κB translocation to the nucleus for cell transcription, exerts an immune response, and produces antibodies [27]. The activation of the IKK/NF-κB signaling pathway can aggravate the clinical symptoms in EAE mice, resulting in the progression of NF-κB-dependent myeloid cell activation and demyelination [28]. In the SLE mouse model, inhibiting IKK and IkB and blocking the activation of the NF-κB pathway can reduce kidney damage in mice [29]. Moreover, knocking out IKKε can reduce the expression of NF-κB, TNF-β, IL-1ε, and IL-6, unveiling a new direction for treating rheumatoid arthritis (RA) [30]. Our results support the abnormal activation of IKK/NF-κB in the acute phase of NMOSD.

CXCL12, which is expressed in various cells, exhibits chemotactic activity for monocytes, neutrophils, and early B-cell precursors [31] and plays an important role in the survival and development of B cells. In the acute phase of demyelination, various inflammatory mediators activate the expression of CXCL12 and stimulate vascular endothelial cells to produce inflammatory chemokines (such as CXCL8) to attract B lymphocytes into the CNS [32]. CXCL12 binds to CXCR4 on the B lymphocyte membrane to promote B lymphocyte BTK Y223 phosphorylation; activate BTK and PI3K; and mediate cell proliferation and adhesion, leukocyte migration, and inflammation [33]. A recent study found that the level of CXCL12 in the peripheral serum of mice with relapsing-relief EAE is elevated, and this is consistent with the continuous peripheral immune response, including the transport and activation of B cells, and may be related to relapse [34]. In our study, the level of CXCL12 in peripheral blood PBMCs of patients in the acute phase of NMOSD was significantly higher than that of the patients in the control group, suggesting that CXCL12 is involved in promoting the inflammatory course of demyelinating in the acute phase of NMOSD. CNS inflammation and increased expression of CXCL12 further amplify the inflammatory cascade. The high expression of CXCL12 in the early stage of MS demyelination can activate the BTK signaling pathway in B lymphocytes and mediate cytotoxicity [35].

## Conclusion

This study showed that BTK, PI3K, IKK, NF-κB, and related inflammatory chemokines CXCL2 and CXCL12 are abnormally activated in the pathogenesis of NMOSD, which can induce B-cell proliferation and differentiation. PI3K is a key factor in the signaling pathway and is highly expressed even in the abnormal activation state of immunity during the remission period in patients with NMOSD. However, the predictive value of PI3K mRNA level for disease recurrence and remission in NMOSD should be further studied in a large sample size to provide a theoretical basis for the pathogenesis and treatment strategy of NMOSD.

## Data Availability

Not applicable.
